# Supplementary figures and images for: Post mortem evaluation of inflammation, oxidative stress, and PPARγ activation in a nonhuman primate model of cardiac sympathetic neurodegeneration
Source: PLoS One. 2020 Jan 7;15(1):e0226999. doi: 10.1371/journal.pone.0226999 (PMC6946159; doi:10.1371/journal.pone.0226999)

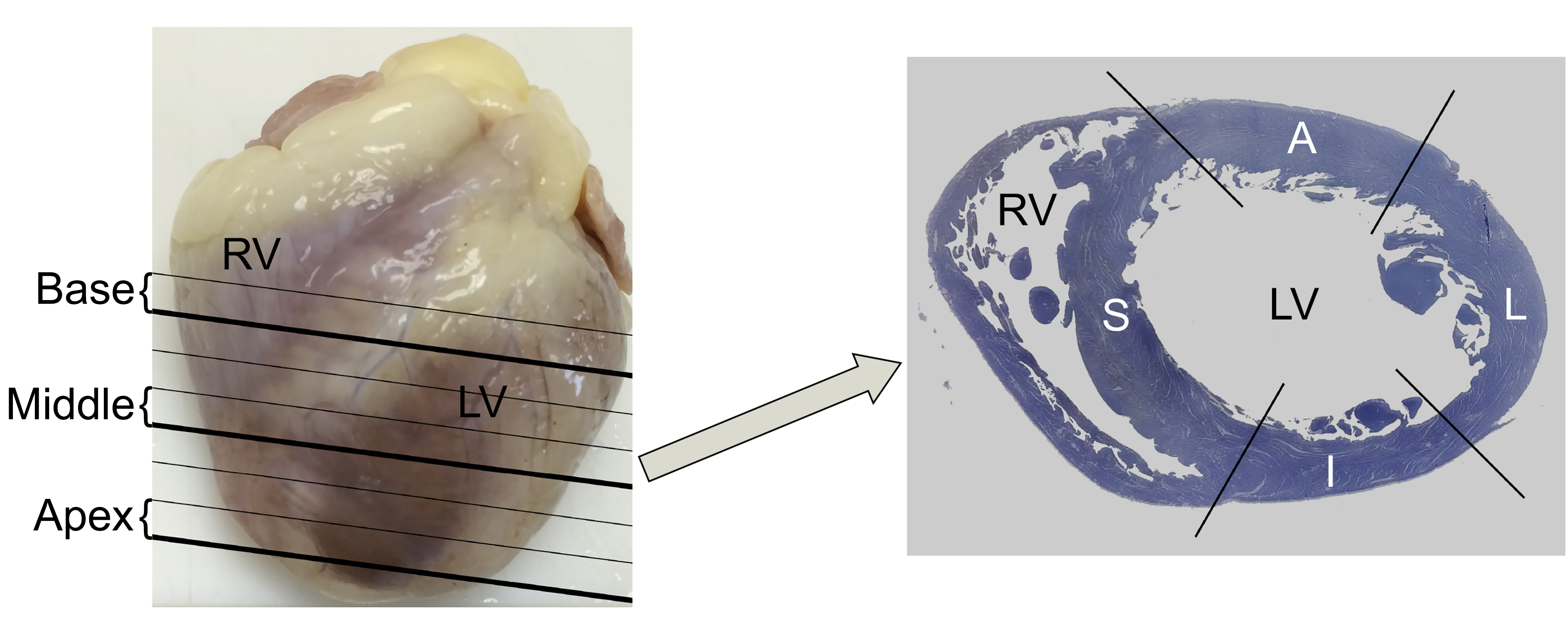

Supplement: S1 Fig — Hearts were collected from each monkey following 4% PFA perfusion. Each heart was post-fixed in 4% PFA and dehydrated in 70% ethanol prior to trimming for paraffin embedding. To make each paraffin embedded block of heart tissue, each heart was cut in transverse sections, starting at the apex, to produce eight 4mm slices representing cardiac levels from the apex to the base. The three levels labeled ‘Apex’, ‘Middle’, and ‘Base’ in the figure were used in this study. Once the tissue from each level was mounted onto slides, it was subdivided into four cardiac regions for analysis: S, septal; A, anterior; L, lateral; I, inferior. RV, right ventricle; LV, left ventricle. The arrow indicates that the example cardiac tissue section in the right of the figure is from the middle cardiac level. (TIF) [file pone.0226999.s001.tif]

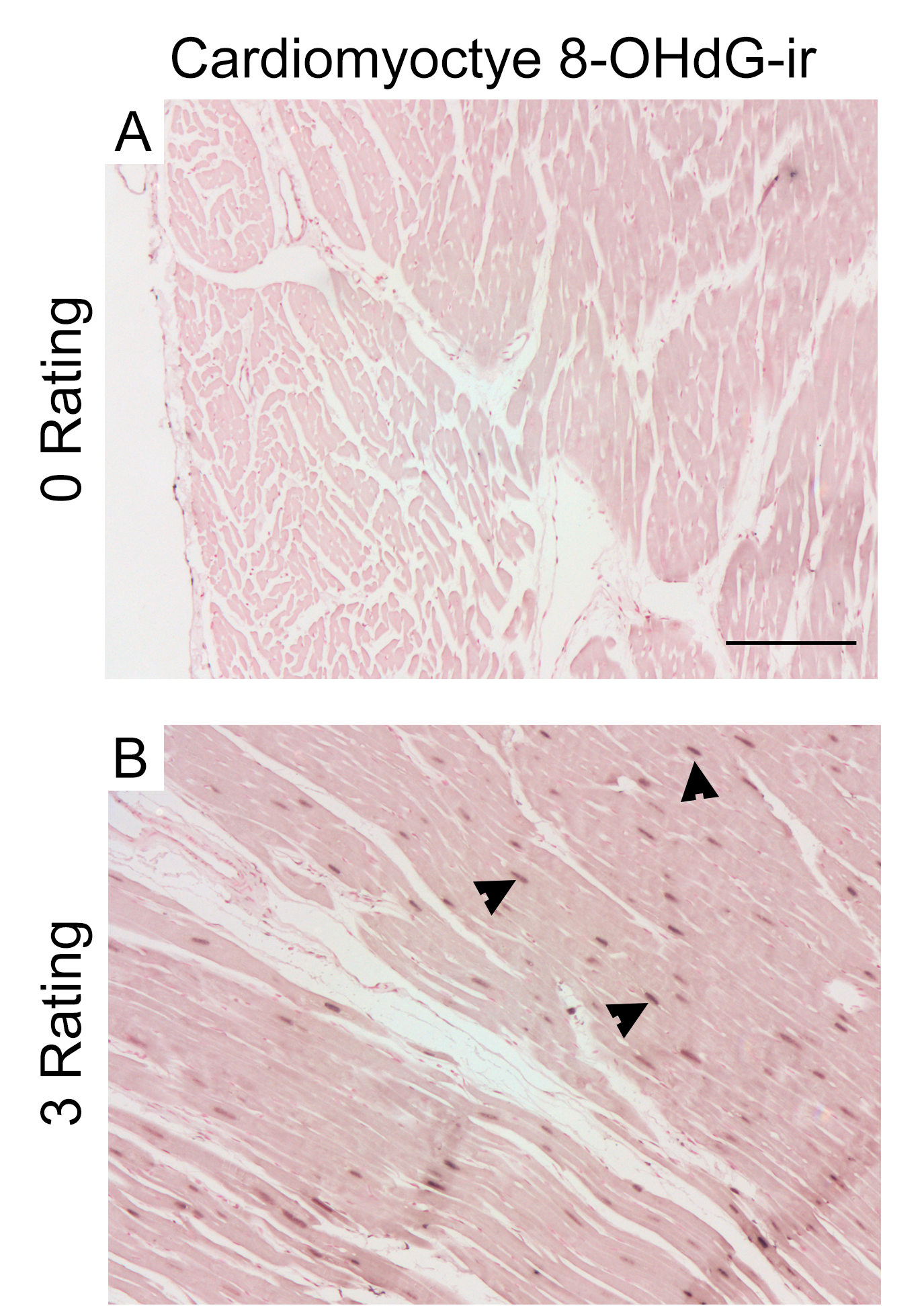

Supplement: S2 Fig — (A,B) Photomicrographs of 8-OHdG immunoreactivity (-ir) in left ventricle cardiomyocytes. Scale bar = 200 μm. (A) Represents a rating of 0 with <10% of cardiomyocyte nuclei immunoreactive for 8-OHdG, while (B) represents a rating of 3 with >50% of cardiomyocyte nuclei immunoreactive for 8-OHdG. The rating scale also included a possible rating of 1 when a diffuse light 8-OHdG stain was observed in 10–100% of cardiomyocyte nuclei OR a medium/dark stain was observed in 10–20% of nuclei and a possible rating of 2 when a medium/dark stain was observed in >20% of nuclei. Black arrowheads point to 8-OHdG-ir cardiomyocyte nuclei. 8-OHdG, 8-hydroxy-2’-deoxyguanosine. (TIF) [file pone.0226999.s002.tif]

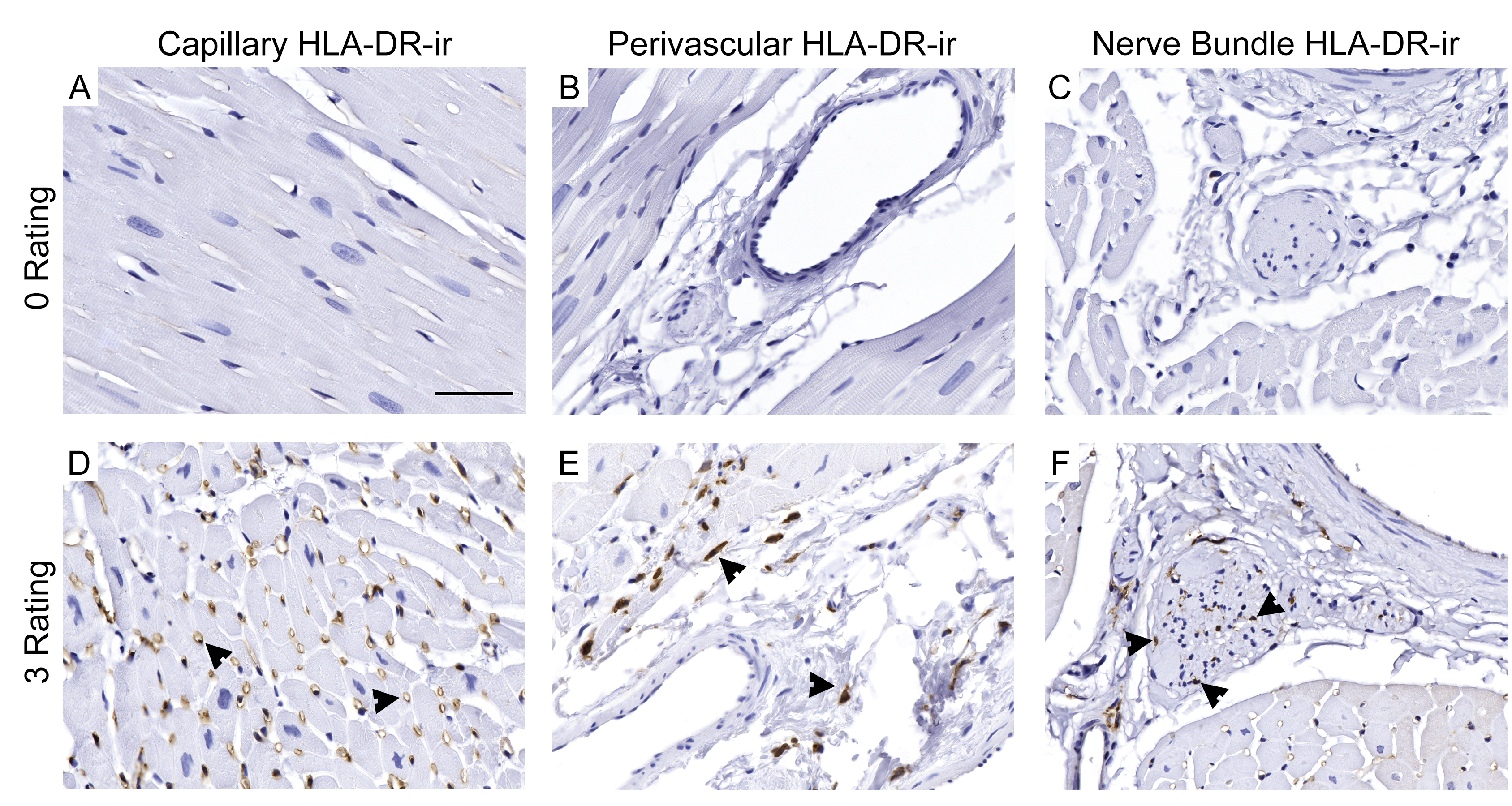

Supplement: S3 Fig — (A-F) Photomicrographs of left ventricle (A, D) capillaries, (B, E) perivascular immune cells, and (C, F) nerve bundles immunostained for the antigen presenting cell marker HLA-DR. Scale bar = 50 μm. (A, B, C) Represent ratings of 0 for each type of HLA-DR immunoreactivity (-ir), while (D, E, F) represent a rating of 3 for each type of HLA-DR-ir. (A, D) Capillary HLA-DR-ir rating is based on the percent area of each cardiac region that exhibited HLA-DR-ir capillaries with a (A) 0 rating having <50% of the region exhibiting HLA-DR-ir capillaries and a (D) 3 rating exhibiting 100% of the region with medium/dark HLA-DR-ir capillaries. The rating scale also included a possible rating of 1 for >50% but less than 100% of the region exhibiting HLA-DR-ir capillaries and a possible rating of 2 for 100% of the region exhibiting light HLA-DR-ir capillaries. (B, E) Perivascular HLA-DR-ir rating is based on the number of HLA-DR-ir cells present within 350 μm of the vessel with a (B) 0 rating having 0 HLA-DR-ir cells and a (E) 3 rating having >10 HLA-DR-ir cells. The rating scale also included a possible rating of 1 for 1 to 5 HLA-DR-ir cells and a possible rating of 2 for 6 to 10 HLA-DR-ir cells. (C, F) Nerve bundle HLA-DR-ir rating is based on the number of HLA-DR-ir cells counted in a nerve bundle with a (C) 0 rating having 0 HLA-DR-ir cells in the bundle and a (F) 3 rating having >5 HLA-DR-ir cells in the bundle. The rating scale also included a possible rating of 1 for 1 or 2 HLA-DR-ir cells in the bundle and a possible rating of 2 for 3 to 5 HLA-DR-ir cells in the bundle. Black arrowheads point to HLA-DR-ir in (D) capillaries, (E) perivascular immune cells, or (F) cells in nerve bundles. HLA-DR, human leukocyte antigen DR. (TIF) [file pone.0226999.s003.tif]

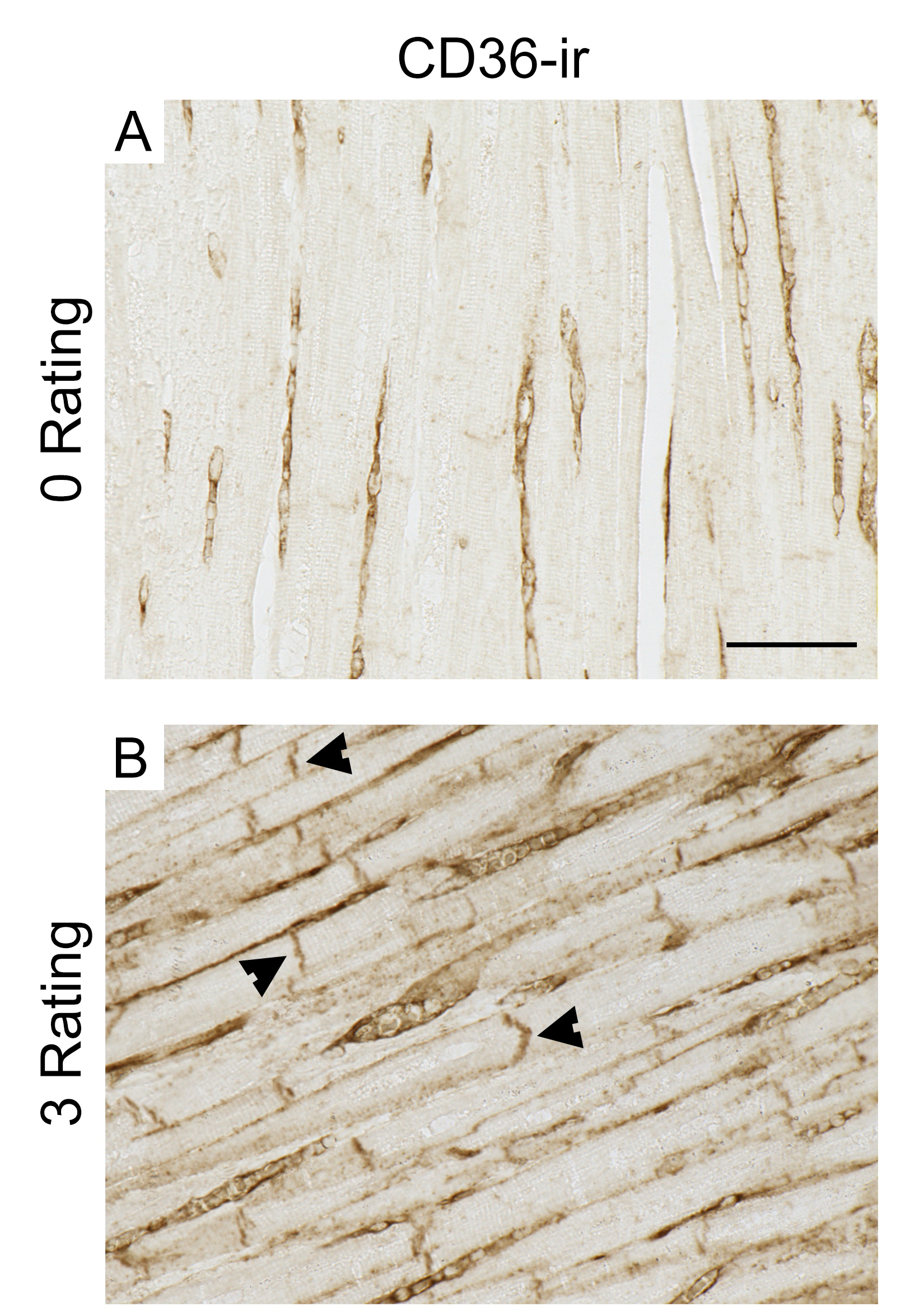

Supplement: S4 Fig — (A,B) Photomicrographs of CD36 immunoreactivity (-ir) in left ventricle cardiomyocytes. Scale bar = 50 μm. (A) Represents a rating of 0 with no to very little CD36-ir at intercalated discs, while (B) represents a rating of 3 with >1/3 of the area of the image showing regularly visible CD36-ir at the intercalated discs. The rating scale also included a possible rating of 1 when CD36-ir was present very lightly in 10–100% of intercalated discs and/or a few (2–4) discs had medium/dark CD36-ir and a possible rating scale of 2 when <1/3 of the area shows regularly visible CD36-ir. Black arrowheads point to CD36-ir intercalated discs. CD36, cluster of differentiation 36. (TIF) [file pone.0226999.s004.tif]

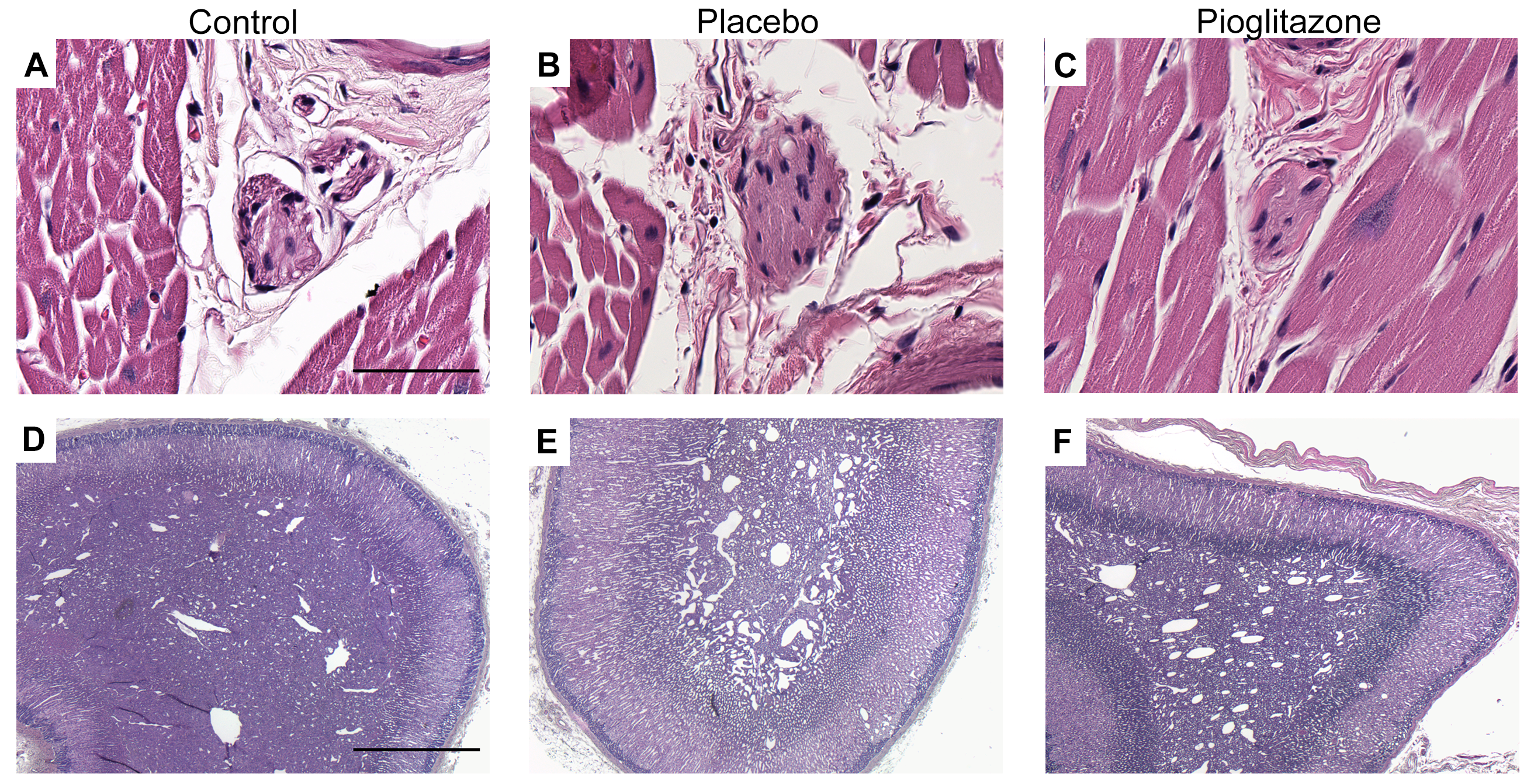

Supplement: S5 Fig — (A-C) Photomicrographs of cardiac left ventricle myocardial nerve bundles, a collection of nerve fibers surrounded by an epineurium, at 63x stained with HE in (A) control, (B) 6-OHDA + placebo, and (C) 6-OHDA + pioglitazone groups. (D-F) Photomicrographs of the adrenal gland at 2.5x stained with HE with medulla visible in the center of the section in (D) control, (E) 6-OHDA + placebo, and (F) 6-OHDA + pioglitazone groups. Scale bar = (A) 50 μm or (D) 2000 μm. 6-OHDA, 6-hydroxydopamine; PPARγ, peroxisome proliferator-activated receptor gamma; HE, hematoxylin and eosin. (TIF) [file pone.0226999.s005.tif]

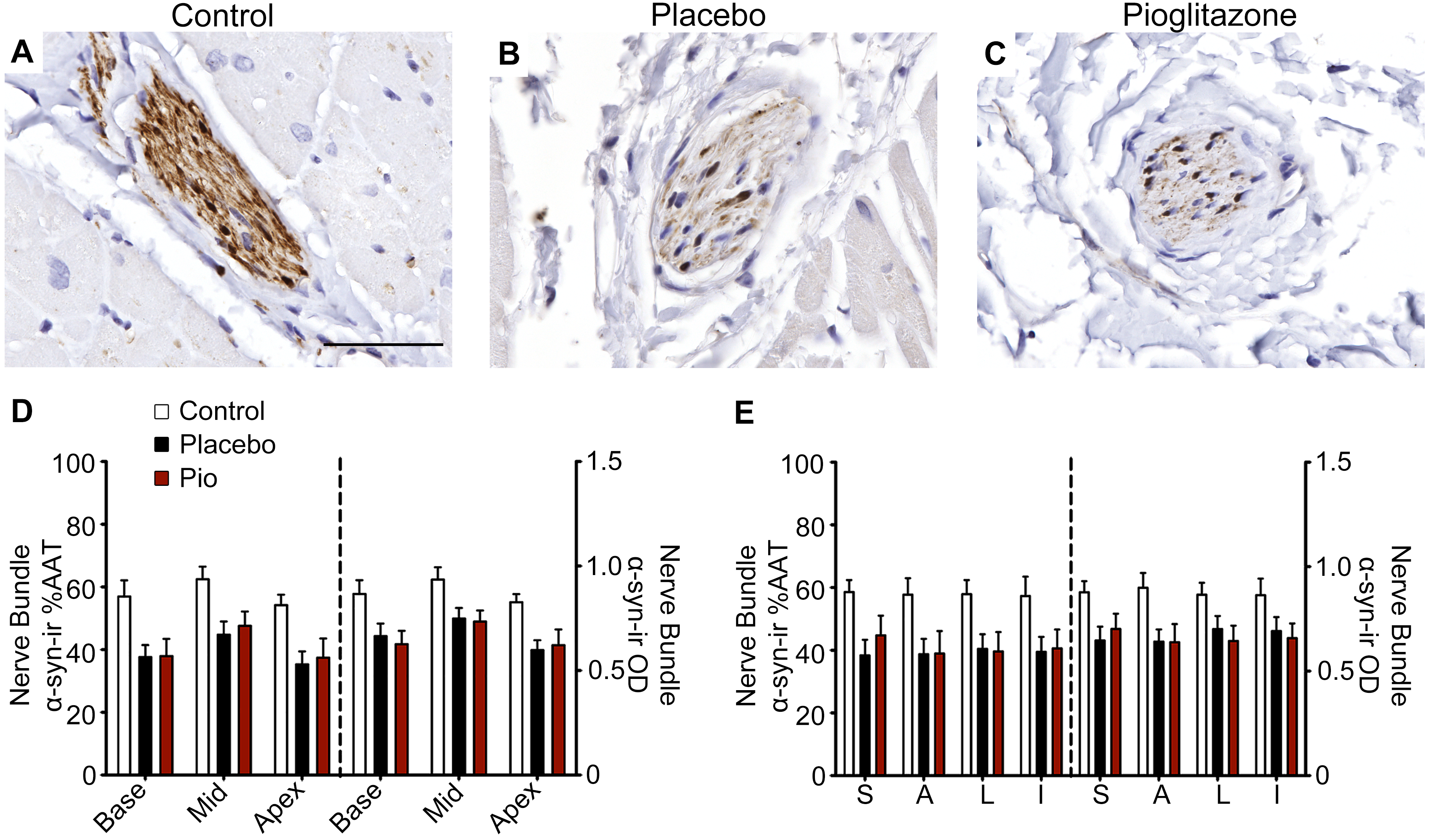

Supplement: S6 Fig — (A-C) Photomicrographs of cardiac left ventricle nerve bundles with α-synuclein (α-syn) immunoreactivity (-ir) in the (A) control, (B) 6-OHDA + placebo, and (C) 6-OHDA + pioglitazone groups. Scale bar = 50 μm. (D) Across all cardiac levels, no statistically significant differences in α-syn-ir %AAT or OD in nerve bundles were found between or within treatment groups. (E) Across all cardiac regions, no statistically significant differences in α-syn-ir %AAT or OD in nerve bundles were found between or within treatment groups. Error bars = SEM. 6-OHDA, 6-hydroxydopamine; PPARγ, peroxisome proliferator-activated receptor gamma; %AAT, percent area above threshold; OD, optical density; Pio, pioglitazone; Mid, middle; S, septal; A, anterior; L, lateral; I, inferior. (TIF) [file pone.0226999.s006.tif]

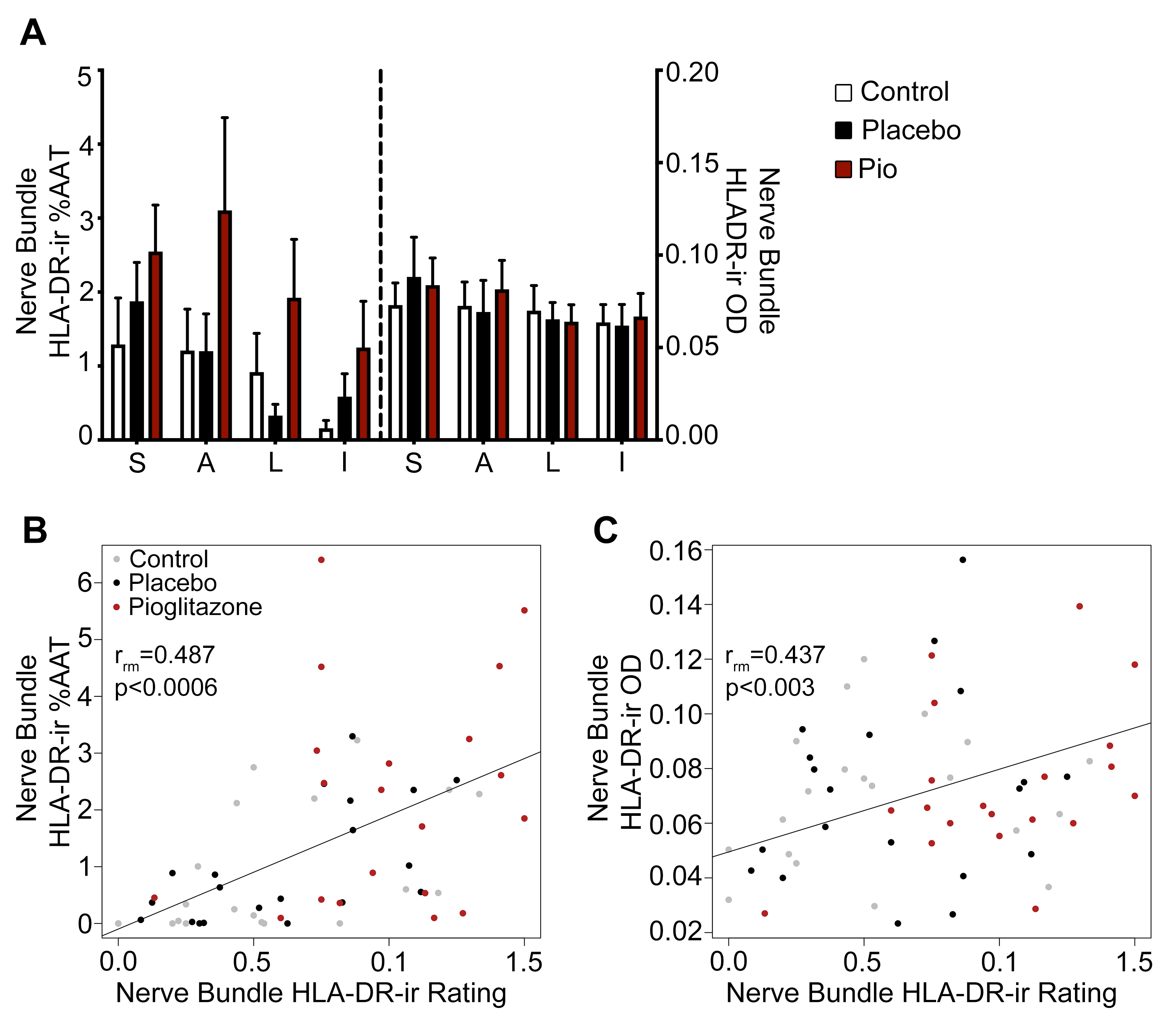

Supplement: S7 Fig — (A) Across cardiac regions, HLA-DR immunoreactivity (-ir) in nerve bundles, as measured by %AAT and OD, was not statistically significantly different between or within treatment groups. Error bars = SEM. (B,C) Plots of repeated measures correlations between nerve bundle HLA-DR-ir semiquantitative ratings and HLA-DR-ir (B) %AAT and (C) OD across cardiac regions in the base level. (B,C) Each point represents a single region in the base level in one animal (4 regions; 15 animals). HLA-DR; human leukocyte antigen DR; Pio, pioglitazone; %AAT, percent area above threshold; OD, optical density S, septal; A, anterior; L, lateral; I, inferior. (TIF) [file pone.0226999.s007.tif]

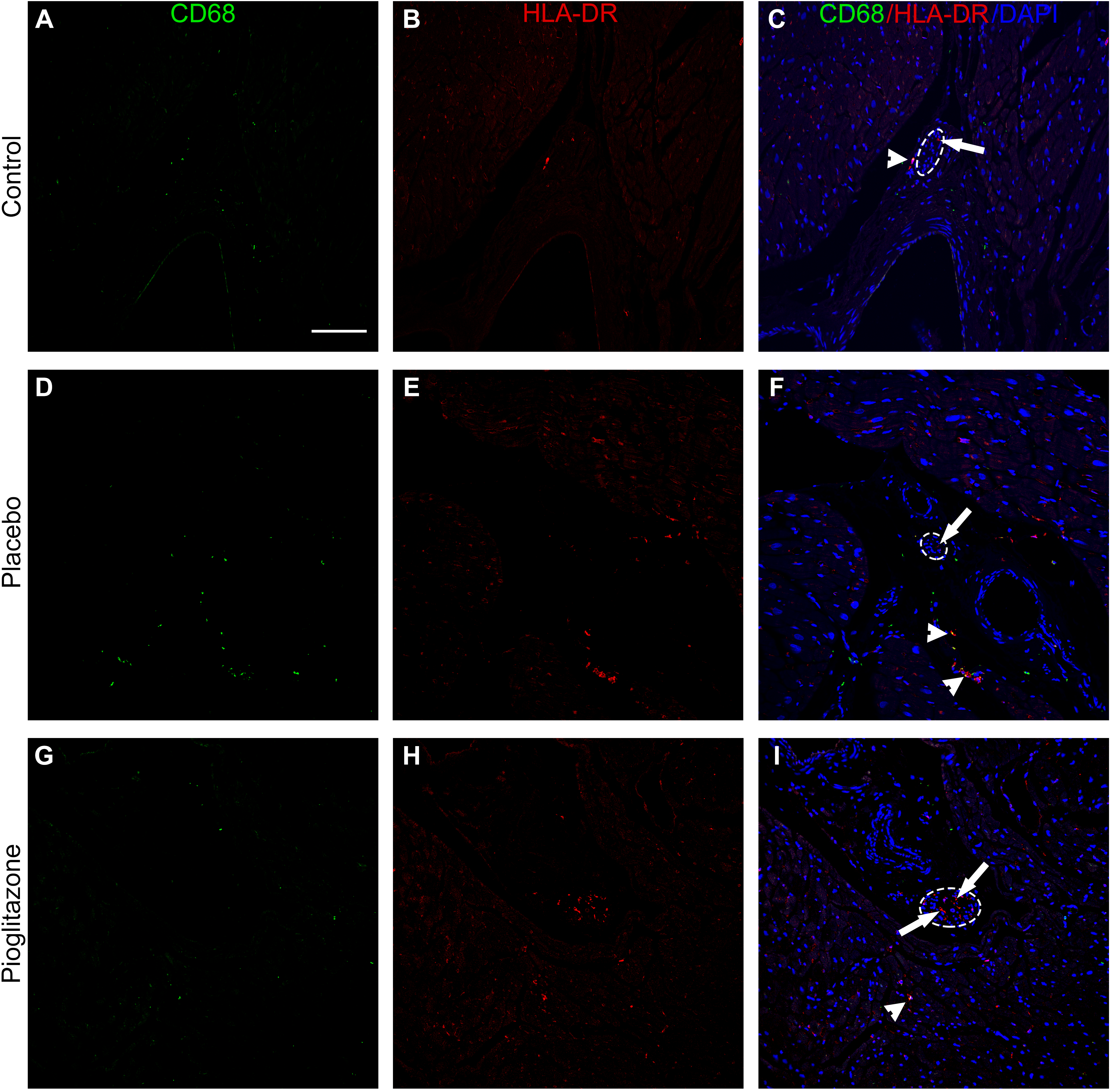

Supplement: S8 Fig — (A-I) Representative photomicrographs of cardiac left ventricle nerve bundles with double-label immunofluorescent labeling of HLA-DR and CD68 in the (A-C) control group, (D-F) 6-OHDA + placebo group, and (G-I) 6-OHDA + pioglitazone group. Note that there are no CD68+ cells in nerve bundles in any group. Scale bar = 100 μm. (C, F, I) Dashed circles outline nerve bundles; white arrows indicate HLA-DR-ir inside nerve bundles; white arrowheads indicate cells outside of nerve bundles that co-label for CD68 and HLA-DR-ir. HLA-DR; human leukocyte antigen DR; CD68, cluster of differentiation 68. (TIF) [file pone.0226999.s008.tif]

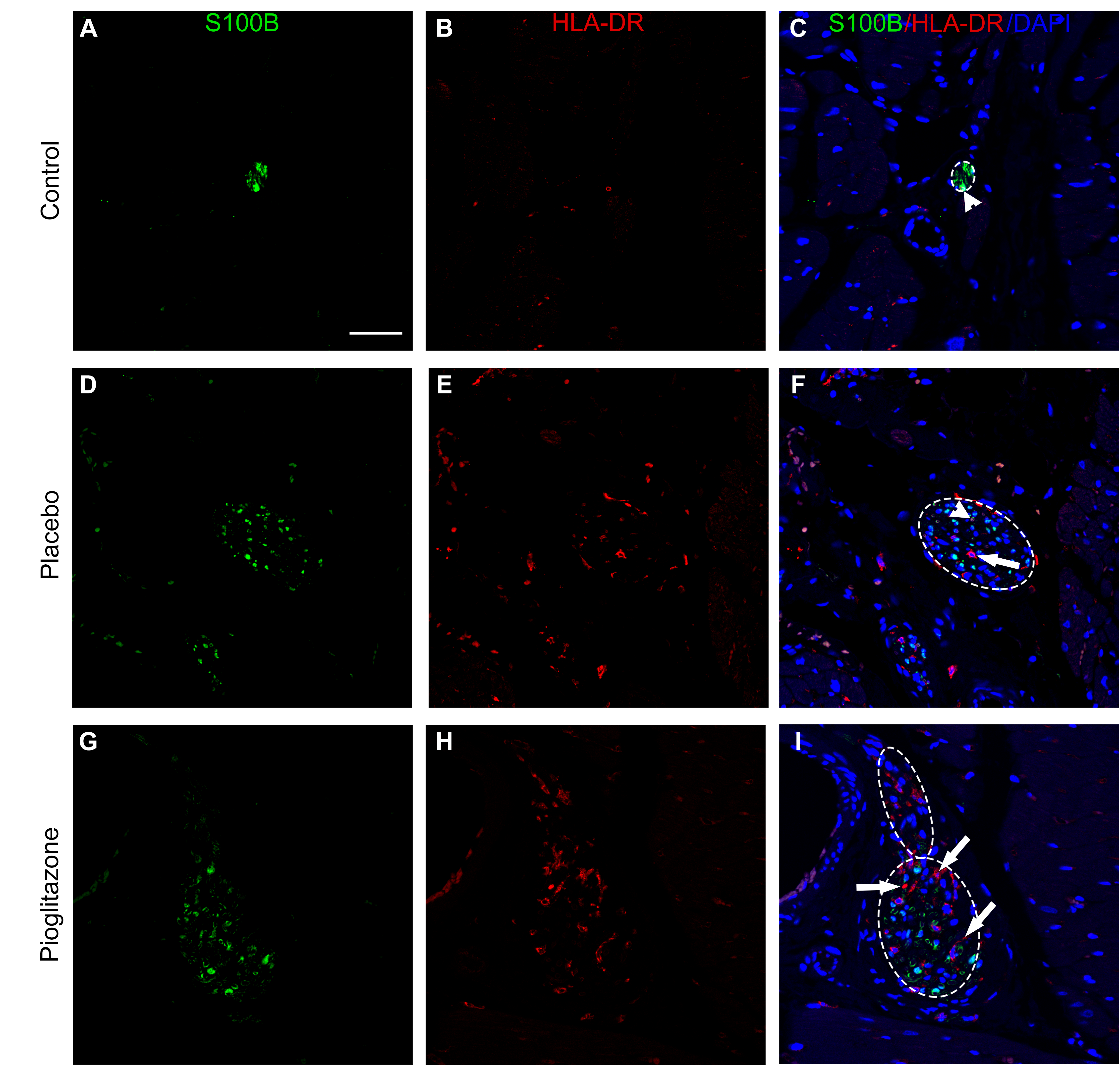

Supplement: S9 Fig — (A-I) Representative photomicrographs of cardiac left ventricle nerve bundles with double-label immunofluorescent labeling of HLA-DR and S100B in the (A-C) control group, (D-F) 6-OHDA + placebo group, and (G-I) 6-OHDA + pioglitazone group. Note minimal HLA-DR/S100B co-labeling in nerve bundles (white arrowheads). Scale bar = 100 μm. (C, F, I) Dashed circles outline nerve bundles; white arrows indicate HLA-DR-ir inside nerve bundles; white arrowheads indicate cells inside of nerve bundles that co-label for S100B and HLA-DR. HLA-DR; human leukocyte antigen DR; S100B, S100 calcium-binding protein B. (TIF) [file pone.0226999.s009.tif]

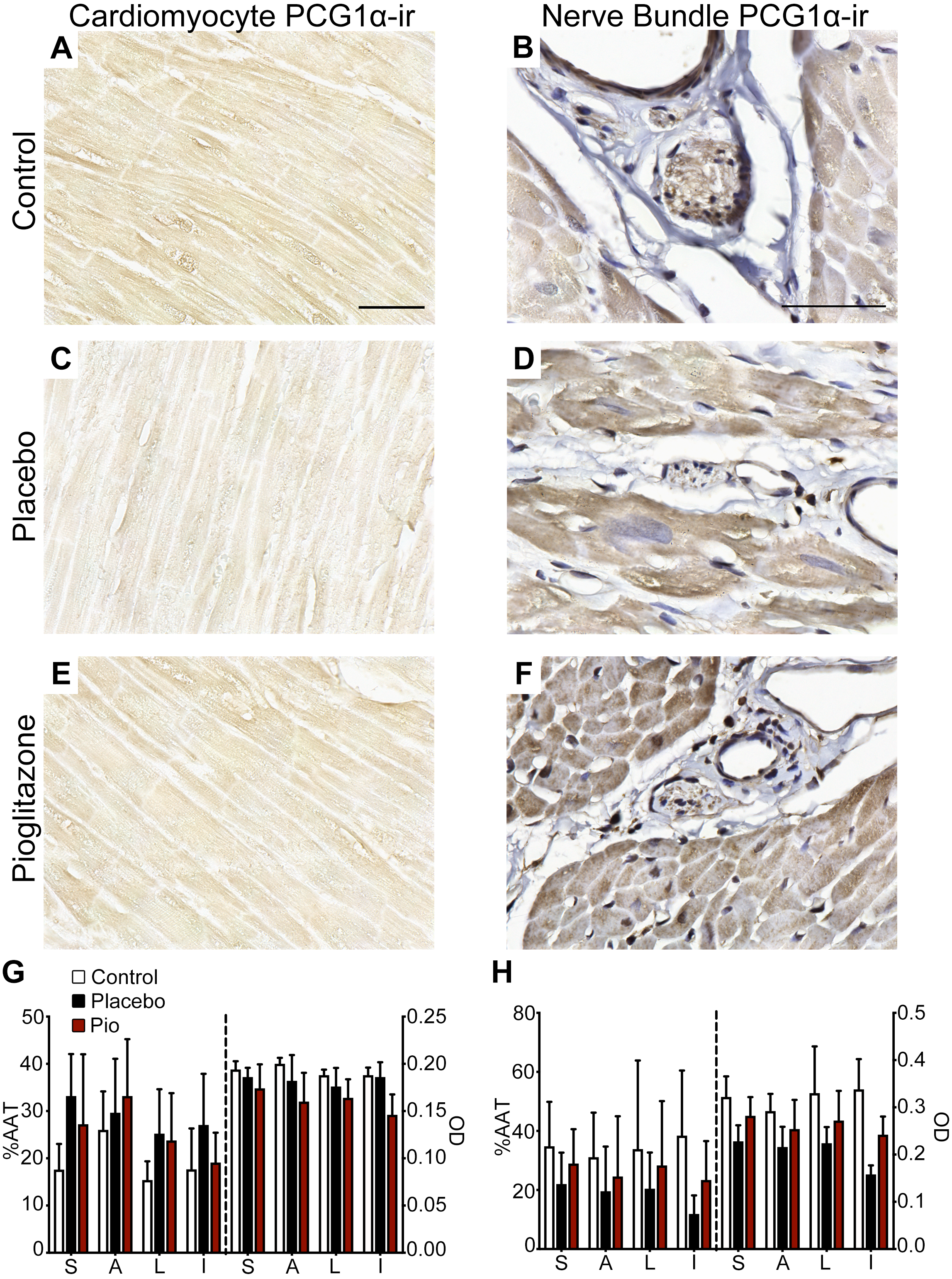

Supplement: S10 Fig — (A-F) Photomicrographs of left ventricle (A, C, E) cardiomyocytes and (B, D, F) nerve bundles immunostained for PGC1α in (A, B) control, (C, D) 6-OHDA + placebo, and (E, F) 6-OHDA + pioglitazone groups. Scale bar = (A, B) 50 μm. (G, H) No differences between or within treatment groups were found for (G) cardiomyocyte PGC1α immunoreactivity (-ir) %AAT or OD or for (H) nerve bundle PGC1α-ir %AAT or OD. Error bars = SEM. 6-hydroxydopamine; PGC1α, peroxisome proliferator-activated receptor gamma (PPARγ) coactivator 1-alpha; Pio, pioglitazone; %AAT, percent area above threshold; OD, optical density; S, septal; A, anterior; L, lateral; I, inferior. (TIF) [file pone.0226999.s010.tif]

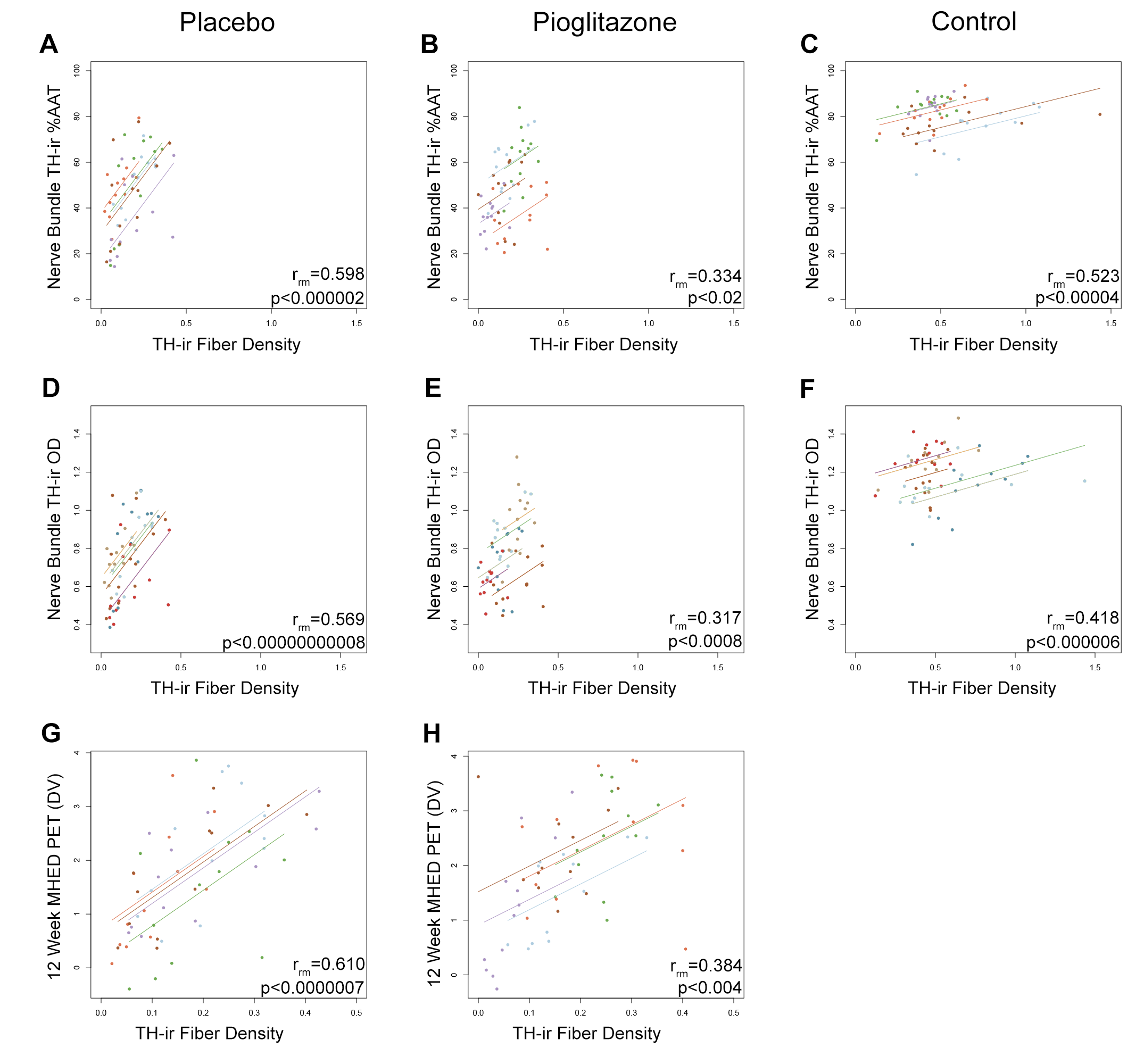

Supplement: S11 Fig — Plots of repeated measures correlations for TH-ir fiber density with (A-C) nerve bundle TH-ir %AAT, (D-F) nerve bundle TH-ir OD, and (G,H) 12 week MHED PET in (A, D, G) 6-OHDA + placebo-treated, (B, E, H) 6-OHDA + pioglitazone-treated, and (C,F) control animals. The dot and line color in each graph represent one animal; note that the same colors are used across treatment groups although these represent different animals in each treatment group. 6-OHDA, 6-hydroxydopamine; TH, tyrosine hydroxylase; %AAT, percent area above threshold; OD, optical density; MHED, [11C]meta-hydroxyephedrine. (TIF) [file pone.0226999.s011.tif]

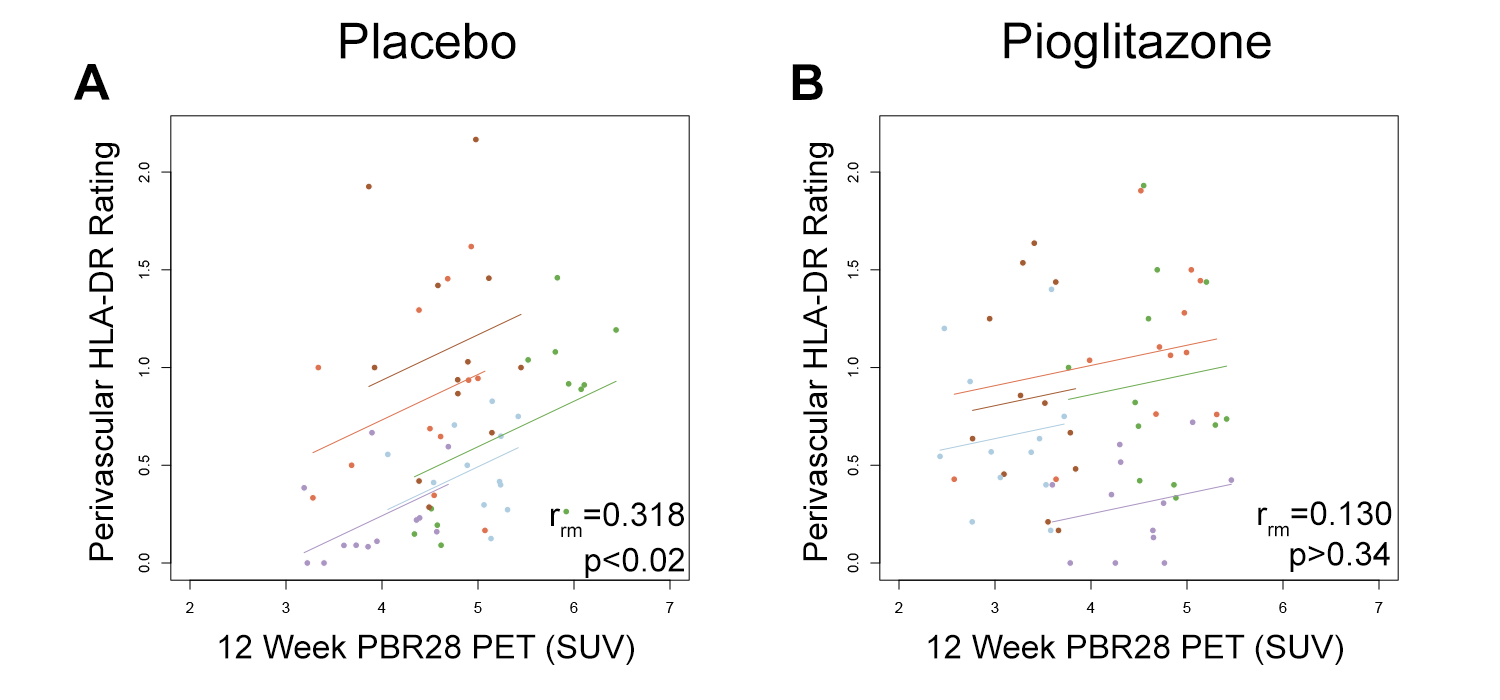

Supplement: S12 Fig — Plots of repeated measures correlations for HLA-DR perivascular semiquantitative rating with 12 week PBR28 uptake in (A) 6-OHDA + placebo- and (B) 6-OHDA + pioglitazone-treated animals. The dot and line color in each graph represent one animal; note that the same colors are used across treatment groups although these represent different animals in each treatment group. 6-OHDA, 6-hydroxydopamine; HLA-DR, human leukocyte antigen DR; PBR28, [11C]PBR28. (TIF) [file pone.0226999.s012.tif]

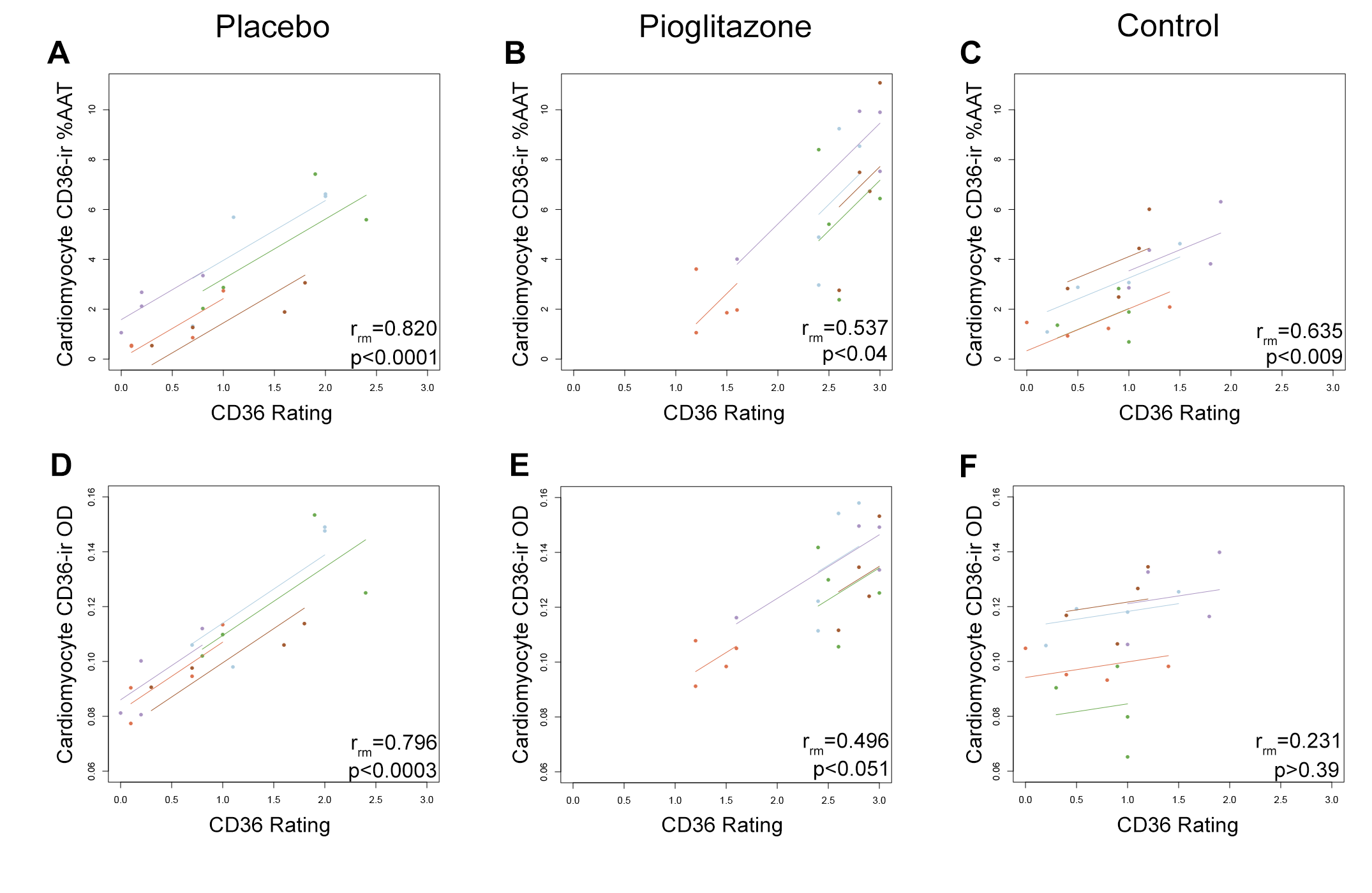

Supplement: S13 Fig — Plots of repeated measures correlations for CD36 rating with (A-C) cardiomyocyte CD36-ir %AAT or (D-F) cardiomyocyte CD36-ir OD in (A,D) 6-OHDA + placebo-treated, (B,E) 6-OHDA + pioglitazone-treated, or (C,F) control animals. The dot and line color in each graph represent one animal; note that the same colors are used across treatment groups although these represent different animals in each treatment group. 6-OHDA, 6-hydroxydopamine; CD36, cluster of differentiation 36; %AAT, percent area above threshold; OD, optical density. (TIF) [file pone.0226999.s013.tif]
