# Supplementary material for: Post mortem evaluation of inflammation, oxidative stress, and PPARγ activation in a nonhuman primate model of cardiac sympathetic neurodegeneration
Source: PLoS One. 2020 Jan 7;15(1):e0226999. doi: 10.1371/journal.pone.0226999 (PMC6946159; doi:10.1371/journal.pone.0226999)
Supplement: S1 Table — (DOCX) [file pone.0226999.s014.docx]

S1 Table. Information about rhesus macaques (*Macaca mulatta*) used in the study.

| **Experimental Group** | **Animal Number** | **Sex** | **Age at Death (yr)** | **Weight at Death (kg)** |
| --- | --- | --- | --- | --- |
| Control | 1 | M | 6.8 | 12.1 |
|  | 2 | M | 12.4 | 12.5 |
|  | 3 | M | 7.8 | 9.7 |
|  | 4 | M | 10.0 | 11.0 |
|  | 5 | M | 6.9 | 9.6 |
| Placebo | 1 | M | 6.6 | 10.9 |
|  | 2 | M | 13.0 | 9.8 |
|  | 3 | M | 9.8 | 12.3 |
|  | 4 | M | 6.9 | 10.1 |
|  | 5 | M | 6.2 | 10.2 |
| Pioglitazone | 1 | M | 11.4 | 10.6 |
|  | 2 | M | 5.6 | 9.4 |
|  | 3 | M | 5.7 | 9.9 |
|  | 4 | M | 6.2 | 10.2 |
|  | 5 | M | 6.2 | 10.5 |

yr, year; kg, kilogram.
