# Supplementary material for: Post mortem evaluation of inflammation, oxidative stress, and PPARγ activation in a nonhuman primate model of cardiac sympathetic neurodegeneration
Source: PLoS One. 2020 Jan 7;15(1):e0226999. doi: 10.1371/journal.pone.0226999 (PMC6946159; doi:10.1371/journal.pone.0226999)
Supplement: S2 Table — (DOCX) [file pone.0226999.s015.docx]

| **Tissue** | **Antigen** | **Marker** | **Company** | **Species** | **Catalog #** | **Lot #** | **Antibody Registry #** | **Dilution** | **Blocking** | **Counterstain** |
| --- | --- | --- | --- | --- | --- | --- | --- | --- | --- | --- |
| Heart | Protein Gene Product 9.5 (PGP9.5) | pan-neuronal, soma and processes | Millipore | rabbit  polyclonal | AB1761 | NG1898214 | AB_91019 | 1:400 | 20% goat serum | Hematoxylin |
| Heart | Tyrosine Hydroxylase (TH) | catecholaminergic | Immunostar | mouse  monoclonal | 22941 | 1241002 | AB_572268 | 1:400 | 20% horse serum | Hematoxylin |
| Heart | Alpha-Synuclein (α-syn) | pre-synaptic protein | Abcam | rabbit  monoclonal | AB138501 | GR154454 | AB_2537217 | 1:200 | 20% goat serum | Hematoxylin |
| Heart | 8-hydroxy-2’-deoxyguanosine (8-OHdG) | oxidative stress | Abcam | Mouse  monoclonal | AB48508 | GR21834-16 | AB_867461 | 1:50 | Super Block solution | Fast Red |
| Heart | Human Leukocyte Antigen DR (HLA-DR) | antigen presenting cells | Dako | mouse  monoclonal | M0746 | 00051226 | AB_2262753 | 1:100 | Super Block solution | Hematoxylin |
| Heart | Cluster of Differentiation 36 (CD36) | Cell surface scavenger receptor/ fatty acid transporter | Sigma | Rabbit  polyclonal | HPA002018 | B1055954 | AB_1078464 | 1:100 | 20% goat serum | None |
| Heart | Peroxisome Proliferator-Activated Receptor (PPAR) Gamma Coactivator 1-Alpha (PGC1α) | Transcriptional coactivator of PPARgamma | NovusBio | Rabbit  polyclonal | NBP1-04676 | G3 | AB_1522118 | 1:100 | Super Block solution | Mix* |
| Adrenal | TH | catecholaminergic | Immunostar | mouse  monoclonal | 22941 | 1241002 | AB_572268 | 1:6000 | Super Block solution | None |
| Adrenal | Aromatic L-Amino Acid Decarboxylase (AADC) | catecholaminergic, serotonergic | Millipore | rabbit  polyclonal | AB1569 | LB1580980 | AB_90789 | 1:2000 | Super Block solution | None |

S2 Table. Primary antibodies used for brightfield immunohistochemistry.

*PGC1α-immunoreactivity in nerve bundles was analyzed in hematoxylin counterstained slides; PGC1α-immunoreactivity in cardiomyocytes was analyzed in non-counterstained slides
