# Supplementary material for: Post mortem evaluation of inflammation, oxidative stress, and PPARγ activation in a nonhuman primate model of cardiac sympathetic neurodegeneration
Source: PLoS One. 2020 Jan 7;15(1):e0226999. doi: 10.1371/journal.pone.0226999 (PMC6946159; doi:10.1371/journal.pone.0226999)
Supplement: S3 Table — (DOCX) [file pone.0226999.s016.docx]

S3 Table. Primary antibodies used for double-label immunofluorescence.

| **Goal of the double IF** | **Antibodies** | **Company** | **Species** | **Catalog #** | **Lot #** | **Antibody Registry #** | **Dilution** | **Blocking Agent** |
| --- | --- | --- | --- | --- | --- | --- | --- | --- |
| Identify potential HLA-DR positive non-myelinating Schwann cells in cardiac nerve bundles | Human Leukocyte antigen DR  (HLA-DR)  & | Dako | mouse monoclonal | M0746 | 0051226 | AB_2262753 | 1:100 | Super Block solution |
|  | S100 calcium-binding protein B  (S100B) | Sigma | rabbit polyclonal | HPA015768 | B80198 | AB_1856538 | 1:800 | Super Block solution |
| Identify potential HLA-DR positive macrophages in cardiac nerve bundles | HLA-DR  & | Dako | mouse monoclonal | M0746 | 0051226 | AB_2262753 | 1:100 | Super Block solution |
|  | Cluster of differentiation 68 (CD68) | Sigma | rabbit polyclonal | HPA048982 | B106831 | AB_2680587 | 1:100 | Super block solution |
