# Supplementary material for: Post mortem evaluation of inflammation, oxidative stress, and PPARγ activation in a nonhuman primate model of cardiac sympathetic neurodegeneration
Source: PLoS One. 2020 Jan 7;15(1):e0226999. doi: 10.1371/journal.pone.0226999 (PMC6946159; doi:10.1371/journal.pone.0226999)
Supplement: S4 Table — (DOCX) [file pone.0226999.s017.docx]

S4 Table. Cardiac tissue histological description.

| **Experimental Group** | **Animal** | **Cardiac Level** | **Histological Findings** | **Diagnosis** |
| --- | --- | --- | --- | --- |
| Control | 1 | Base | Fatty infiltration into myocardium | none |
|  |  | Middle | Mild chronic interstitial change |  |
|  |  | Apex | n/a |  |
|  | 2 | Base | Obesity associated adipocyte infiltration | none |
|  |  | Middle | Obesity associated adipocyte infiltration |  |
|  |  | Apex | Obesity associated adipocyte infiltration |  |
|  | 3 | Base | n/a | none |
|  |  | Middle | n/a |  |
|  |  | Apex | n/a |  |
|  | 4 | Base | n/a | none |
|  |  | Middle | n/a |  |
|  |  | Apex | n/a |  |
|  | 5 | Base | n/a | none |
|  |  | Middle | Fatty infiltration in the myocardium |  |
|  |  | Apex | Fat at apex and in myocardium |  |
| Placebo | 1 | Base | Tiny aggregate of lymphocytes in papillary muscle and in right ventricle; lymphocyte inflammation in septum (mild, multifocal) | Minimal lymphocytic myocarditis |
|  |  | Middle | n/a |  |
|  |  | Apex | Minimal foci of lymphocytic infiltration in myocardium |  |
|  | 2 | Base | Minimal degenerative and fibrosing cardiomyopathy where septum joins ventricular wall | none |
|  |  | Middle | Minimal degenerative and fibrosing cardiomyopathy in papillary muscle |  |
|  |  | Apex | n/a |  |
|  | 3 | Base | Mild fibrosis in papillary muscles^#^; minimal fibrosis in ventricular free wall | none |
|  |  | Middle | Minimal fibrosis in papillary muscles^#^ |  |
|  |  | Apex | n/a |  |
|  | 4 | Base | n/a | Minimal focal neutrophilic myocarditis |
|  |  | Middle | Minimal fibrosis (interstitial fibrosis) within papillary muscle |  |
|  |  | Apex | Very small aggregate of neutrophils (about 7) in lateral free wall |  |
|  | 5 | Base | Minimal fibrosis in papillary muscles and in right ventricle inferior^#^ | none |
|  |  | Middle | Minimal fibrosis in papillary muscles and in right ventricle inferior^#^ |  |
|  |  | Apex | n/a |  |
| Pioglitazone | 1 | Base | n/a | none |
|  |  | Middle | Minimal subepicardial degenerative and fibrosing cardiomyopathy |  |
|  |  | Apex | n/a |  |
|  | 2 | Base | Minimal fibrosis extending slightly into margins of some papillary muscles^#^; minimal lymphocytic perivascular cuff | none |
|  |  | Middle | n/a |  |
|  |  | Apex | n/a |  |
|  | 3 | Base | Minimal fibrosis in papillary muscle^#^ | none |
|  |  | Middle | n/a |  |
|  |  | Apex | Subacute, minimal focal myocardial degeneration with no fibrosis |  |
|  | 4 | Base | Mild to moderate subepicardial fibrosis; adipocyte infiltration into myocardium | 1) minimal multifocal lymphocytic myocarditis 2) mild to moderate degenerative and fibrosing cardiomyopathy 3) mild multifocal adipocyte infiltration |
|  |  | Middle | Anteroseptal focus of interstitial fibrosis with degeneration and cardiomyocyte loss |  |
|  |  | Apex | Small area of fibrosis; two small foci of mild lymphocytic infiltration |  |
|  | 5 | Base | n/a | none |
|  |  | Middle | n/a |  |
|  |  | Apex | n/a |  |

n/a, no significant histological findings; none, histological findings are unremarkable and do not warrant a diagnosis; ^#^, typical background finding.
