# Supplementary material for: Post mortem evaluation of inflammation, oxidative stress, and PPARγ activation in a nonhuman primate model of cardiac sympathetic neurodegeneration
Source: PLoS One. 2020 Jan 7;15(1):e0226999. doi: 10.1371/journal.pone.0226999 (PMC6946159; doi:10.1371/journal.pone.0226999)
Supplement: S5 Table — (DOCX) [file pone.0226999.s018.docx]

S5 Table. Adrenal tissue histological description.

| **Experimental Group** | **Animal ID** | **Histological Findings** |
| --- | --- | --- |
| Control | 1 | n/a |
|  | 2 | n/a |
|  | 3 | Some mineralization^1^; small focus of extracapsular adenomatous hyperplasia^2^ |
|  | 4 | n/a |
|  | 5 | 3 small foci of extracapsular adenomatous hyperplasia^2^ |
| Placebo | 1 | Large amount of mineralization^1^; some fibrosis |
|  | 2 | n/a |
|  | 3 | n/a |
|  | 4 | n/a |
|  | 5 | n/a |
| Pioglitazone | 1 | Mild to moderate lymphocytic medullary infiltration with moderate medullary cell atrophy, vacuolization, and loss. Tyrosine hydroxylase (TH) immunohistochemistry revealed medullary cells with diminished to absent staining. |
|  | 2 | Small focus of extracapsular adenomatous hyperplasia^2^ |
|  | 3 | n/a |
|  | 4 | n/a |
|  | 5 | n/a |

Notes: n/a, no notable histological findings; ^1^, multifocal mineralization is an occasional, incidental finding in rhesus macaques; ^2^, extracapsular adenomatous hyperplasia is a common finding in rhesus, especially with age.
