# Supplementary material for: Post mortem evaluation of inflammation, oxidative stress, and PPARγ activation in a nonhuman primate model of cardiac sympathetic neurodegeneration
Source: PLoS One. 2020 Jan 7;15(1):e0226999. doi: 10.1371/journal.pone.0226999 (PMC6946159; doi:10.1371/journal.pone.0226999)
Supplement: Attachment — Submitted filename: renamed_9bc58.docx [file pone.0226999.s022.docx]

Neuroanatomical effects of PPARγ activation in a nonhuman primate model of cardiac sympathetic neurodegeneration

The dysautonomia’s model proposed by the authors is the systemic 6OHDA injection and its effects upon cardiac cathechoalminergic inervation and adrenal medulla cromaffin cell integrity. Oxidative stress investigated and inflamation as trigher of cellular loss was investigated. Neuroprotective effect of pioglitazone an anti-diabetic drug was also analysed.

The aim of the present study is relevant to the Parkinson’s disease physiopathology. Parkinsonism is much more a syndrome than a disease. The methodology is in accordance with the aim of the study, statistical analysis utilized very robust and the results can help to shed light to early parkinsonism diagnosys and treatment of non motor effects.

I have one question to the authors, which does not invalidade the presente model, my question is:

Considering that parkinsonism has its damage in substantia nigra, a mesencephalic structure, with death of dopaminergic mesostriatal fibres. The cause or trigger this cellular death is still unkwon from its description, 1817 till now. In the model presented in your paper, cathechoalminergic impairment is caused by systemic 6OHDA injection, which pathway in a parkinsoniam patient you see similarities? Are your model suggesting that maybe parkinsonism does not have as main inicial target dopaminergic nigral cells or saying in another way could the disease or syndrome starts in cathechoalmiergic peripheral terminal and progresses to central neurons? If so which intermediate of cathechoalmiergic catabolism could be the vilan?
