# Supplementary material for: Post mortem evaluation of inflammation, oxidative stress, and PPARγ activation in a nonhuman primate model of cardiac sympathetic neurodegeneration
Source: PLoS One. 2020 Jan 7;15(1):e0226999. doi: 10.1371/journal.pone.0226999 (PMC6946159; doi:10.1371/journal.pone.0226999)
Supplement: Attachment — Submitted filename: Metzger_LetterResponsetoReviewersRoundTwo_ PLOS_12062019.doc [file pone.0226999.s024.doc]

**Please find in the following pages the referee comments quoted in italics. Our responses follow each one of the queries.**

“*Reviewer #2: Metzger and colleagues have adequately addressed all of my comments. Indeed, the authors made a very good job; each question was clearly answered, and the authors have explained their methods (particularly, the statistical analyses used) in detail. Congratulations!”*

<< We thank the reviewer for appreciating the updates we made to the manuscript and our responses to the reviewer’s questions. >>

“*I have only two suggestions as follows.*

*Methods, line 113. Please report the data regarding ambient temperature in Celsius degree, as reported for the incubation temperature (line 198).*”

<< Great catch! We have updated this line with the correct room temperature in degrees Celsius. >>

“*Methods, lines 366 and 367. Please replace “normality using R using the qq plot feature” with “normality using the qq plot feature of R software”.*”

<< We have updated the manuscript as suggested. >>
